# Care Plan Trifecta: A Multidisciplinary Approach for Person‐Centered Care Planning for Persons Living with a Dementia Diagnosis of AD/ADRD and Caregivers

**DOI:** 10.1002/alz70858_106479

**Published:** 2025-12-25

**Authors:** Katherine Brandt, Amy M Almeida, Brad C. Dickerson

**Affiliations:** ^1^ Massachusetts General Hospital Frontotemporal Disorders Unit, Boston, MA, USA; ^2^ MGH Frontotemporal Disorders Unit, Boston, MA, USA; ^3^ Frontotemporal Disorders Unit and Massachusetts Alzheimer's Disease Research Center, Department of Neurology, Massachusetts General Hospital and Harvard Medical School, Boston, MA, USA

## Abstract

**Background:**

Persons living with a diagnosis (PLWD) of Alzheimer's disease or related dementia (AD/ADRD) and their caregivers (CGs) commonly report feeling unsure of next steps after receiving a diagnosis. Clinicians are viewed as trusted experts to provide guidance for care planning but often have limited time for care management and insufficient knowledge of PLWD needs outside of medical management.

**Method:**

As a part of a larger Caregiver Support Program offered through the Massachusetts General Hospital (MGH) Frontotemporal Disorders (FTD) Unit, a framework was developed to organize the issues that should be considered in care planning. The Care Plan Trifecta (CPT) provides a framework for person‐centered care planning that identifies specific needs of the PLWD and CGs, prioritizes personhood and dignity, balances independence with safety, and recognizes goals of care at every stage. CPT focuses on three domains considered from the perspective of communities: medical, home and patient advocacy. Medical community emphasizes an interdisciplinary medical team and communication between providers to promote continuity of care. Home community encourages connections with local service providers for legal, financial, and activity planning, community‐based programs to access adult day health programs, in‐home personal care, respite and memory care. Patient advocacy community provides support groups, education and advocacy opportunities to propel funding and social policy for AD/ADRD research, care and services forward.

**Result:**

The CPT has been integrated into the MGH FTD Unit caregiver support program through one‐on‐one counseling sessions that encourage early care planning related to a variety of topics including advance care planning with consideration for individual values and culture. The CPT has been presented to family caregivers, community providers and professional leaders in the field of dementia care through online webinars, in‐person family education events and scientific conferences.

**Conclusion:**

Persons living with a diagnosis of AD/ADRD and caregivers benefit from guidance about the elements of care planning that incorporate social and community resources beyond medical care. Clinicians and healthcare providers benefit from a framework that can be personalized to meet the unique lenses through which each PLWD and family experience their journey, including personal and family values, spiritual, cultural, geographic, and socioeconomic considerations.

